# Notes and News

**Published:** 1898-12-31

**Authors:** 


### NOTES AND NEWS.

(Collected and Communicated.)

The new Kingston Hospital, the Jubilee offering of the people of Kingston, has been formally opened by the Duke of Cambridge.

The foundation-stone of the new Mater Infirmorum Hospital at Belfast has been laid by the Most Rev. Dr. Henry. It is anticipated that the work will be completed in June.

The Council of the After-Care Association for Poor Persons Discharged Recovered from Asylums for the Insane appeal for help to carry on and extend their work. Contributions will be thankfully received by the secretary, Mr. H. Thornhill Roxby, Church House, Dean's Yard, Westminster, S.W., or they may be paid into the account of the association at the Union Bank of London, Argyll Place, London, W. The Worshipful Company of Clothworkers have already made a donation of £10 to the funds.

The United States army is to have considerable additions made to its hospital accommodation. Sixty portable hospitals are under order. Each is to contain 40 patients. They are to be 121 ft. by 19 ft. 3 in., to be constructed in sections, and to have 44 windows and five doors. Ventilation by means of outlets in the ceiling is also arranged. They are to stand 18 in. from the ground, and to be constructed of North Carolina pine, with roofing of painted canvas. The hospitals for use in the north are to have double floors with felt linings.

The British National Society for Aid to the Sick and Wounded in War, or, in shorter terms, the British Red Cross Society, has just issued a report of its operations in connection with the Turco-Greek war, 1897-98, and in the Soudan Expedition, 1898. It appears that an amount exceeding £5,600 was expended by the society in connection with the Turco-Greek war, of which sum about £3,100 was expended in Greece and £2,500 in Turkey. The amount spent in the Soudan Expedition was £2,726, including £500 to the sick and wounded of the Egyptian army and the dervish wounded at Omdurman, and £1,014 for the expenses in connection with the hire of the hospital ship "Mayflower."

A patient suffering from the plague was landed at Plymouth from the "Golconda," from Calcutta, on Saturday, having shown decided symptoms of the disease when the ship left Marseilles. The patient is an officer in the British India Company's service, and has only a mild attack of the plague. Isolation was carefully carried out on board, and all proper precautions as to the ship were taken at Plymouth and in the Thames.

The Hon. Sydney Holland, chairman of the London Hospital, is issuing an appeal to 365 wealthy persons, asking them to contribute £200 each to the institution on their birthdays. We learn that he has already received several favourable replies to his request. The *Sun* newspaper is also working in aid of the London Hospital, for which it asks of its readers 400 soft pillows, to replace a like number now in use at the hospital which are not feather pillows.

An urgent appeal is now being made for the sum of £12,000 for the purpose of completing the British Home for Incurables by building an entertainment hall, and further accommodation for patients and staff. Towards this amount a donation of £1,000 has already been received from a lady who wishes to endow a bed in the new wing in memory of her mother. Donations are also required for the purpose of filling in the east window of the chapel with stained glsss, the present white light being very trying to the eyesight of the patients attending the services. Cheques and postal orders should be crossed "Barclay and Co.," and sent to R. G. Salmond, Secretary, 72, Cheapside, E.C.

Mr. Woolf Joel bequeathed no less than £25,000 to the London charities, and the following sums have accordingly been distributed by Messrs. Barnato Brothers amongst the London hospitals: £1,050 each to Middlesex Hospital and West London Hospital; £1,000 each to Charing Cross Hospital, London Hospital, St. Mary's Hospital, St. Thomas' Hospital, Westminster Hospital, and Metropolitan Hospital; £500 each to Cancer Hospital, City Orthopædic Hospital, Evelina Hospital for Sick Children, Hospital for Consumption and Diseases of the Chest (Brompton), Hospital for Women, National Hospital for the Paralysed and Epileptic, North-Eastern Hospital for Children, Queen Charlotte's Lying-in Hospital, Royal London Ophthalmic Hospital, City of London Hospital for Diseases of the Chest, and St. George's Hospital; £250 each to Alexandra Hospital for Children with Hip Disease, Hospital and Home for Incurable Children, National Orthopædic Hospital, Paddington Green Children's Hospital, Public Dispensary (Clare Market), Samaritan Free Hospital, Seamen's Hospital (Dreadnought), Surgical Aid Society, and Victoria Hospital for Children; £100 each to Central London Hospital, French Hospital and Dispensary, London Lock Hospital and Rescue Home, Poplar Hospital for Accidents, Royal Hospital for Diseases of the Chest, and Royal Westminster Ophthalmic Hospital; £50 each to Central London Throat, Nose, and Ear Hospital, Hospital for Epilepsy and Paralysis, London Homœopathic Hospital, Royal Hospital for Children and Women, Royal Orthopædic Hospital, St. Peter's Hospital, and St. Saviour's Hospital and Nursing Home.

